# Phenolic Acid Distribution in Wheat Pearling Fractions Using Microwave-Assisted Extraction

**DOI:** 10.3390/foods15101828

**Published:** 2026-05-21

**Authors:** Kemashalini Kirusnaruban, Nicola Gasparre, Ruchira Nandasiri, Michael N. A. Eskin, Cristina M. Rosell

**Affiliations:** 1 Department of Food and Human Nutritional Sciences, University of Manitoba, Winnipeg, MB R3T 2N2, Canada; ketheesk@myumanitoba.ca (K.K.); nicola.gasparre@umanitoba.ca (N.G.); hewa.nandasiri@umanitoba.ca (R.N.); michael.eskin@umanitoba.ca (M.N.A.E.); cristina.rosell@umanitoba.ca (C.M.R); 2 St. Boniface Hospital Research Centre, Winnipeg, MB R2H 2A6, Canada; 3 Institute of Agrochemistry and Food Technology (IATA-CSIC), Paterna 46980, Spain

**Keywords:** wheat, phenolic acids, pearled fractions, microwave-assisted extraction, total phenolic content

## Abstract

Phenolic acids are bioactive compounds in wheat (*Triticum aestivum* L.) that contribute to its nutritional and functional properties, yet their distribution within the kernel is uneven. This study investigated the effect of progressive pearling on phenolic acid distribution using microwave-assisted extraction (MAE) with water as a solvent. Three commercial Canada Western Red Spring wheat samples were pearled into six fractions (50–450 s), corresponding to 5–45% removal of outer kernel layers. Pearled kernels, pearled kernel flours, and pearled fractions were analyzed for total phenolic content (TPC) and individual phenolic acids using HPLC-DAD. The 10% pearled fraction (PF100) exhibited the highest TPC (9286 ± 168 µg GAE/g), confirming phenolic enrichment in the outer bran and sub-aleurone layers. Outer kernel tissues contained the highest gallic acid (1954 µg/g), whereas the endosperm retained lower levels of gallic (450 µg/g), hydroxycinnamic (122 µg/g), sinapic (87 µg/g), and ferulic (84 µg/g) acids. Both TPC and individual phenolic acids decreased progressively with increased pearling depth, indicating a clear localization gradient. MAE with water enhanced extraction efficiency compared to conventional solvent-based methods, enabling environmentally friendly recovery. These findings demonstrate that controlled pearling can be used to enrich wheat fractions in phenolic acids and optimize functional ingredient development.

## 1. Introduction

Wheat (*Triticum aestivum* L.) is one of the most important cereal crops worldwide and serves as a major source of carbohydrates, proteins, vitamins, and minerals for millions of people [1,2]. The outer layers of the wheat grain, especially the bran and aleurone, are rich in phenolic acids, which contribute to the nutritional and functional quality of wheat-based foods [3,4]. The health benefits of dietary phenolic acids, including their antioxidant and disease-preventive properties, have been widely reported [5]. However, during conventional milling, most of these outer layers are removed. This results in substantial losses of phenolic compounds, which are mainly concentrated in these tissues [6,7]. Processing techniques such as pearling can increase these losses, resulting in pearled kernel flours with significantly reduced phenolic content [8,9]. Longer pearling durations generally produce more highly pearled flours, and greater reductions in phenolics, ultimately influencing the nutritional quality of wheat-based products [10]. Understanding how processing affects phenolic retention is therefore essential for improving the health value of wheat products.

Although considerable research has focused on phenolic compounds in whole wheat, relatively few studies have examined how controlled pearling intensity influences the phenolic acid profile of intact wheat kernels. Early work by Beta et al. [11] evaluated total phenolic content in different pearled fractions, while Sovrani et al. [12] described the distribution of free phenolic acids among incremental pearled fractions of commercial winter wheat. Zhang et al. [13] characterized phenolic acids in distinct bran components of pigmented wheat, and Spaggiari et al. [14] quantified free and bound phenolics in flour and milling by-products. More recently, Tian et al. [15] examined processing effects on phenolic composition using pearled flour, pearled fractions, and whole grain.

Despite these advances, no study has systematically evaluated the phenolic acid profile of whole wheat kernels subjected to progressive pearling intensities as a controlled fractionation approach. In the present study, pearling up to 45% was applied as an experimental tool to progressively remove outer kernel layers and establish a phenolic distribution gradient across kernel depth. This approach was not intended to simulate commercial milling conditions, but rather to better understand the relative contribution of outer and inner kernel regions to overall phenolic acid content.

In addition, most previous investigations have relied on conventional solvent-based extraction techniques, which are time-consuming and require substantial volumes of organic solvents, raising environmental and safety concerns. Microwave-assisted extraction (MAE) offers a faster, more energy-efficient, and potentially solvent-reduced alternative for recovering phenolic compounds. MAE has shown promising results for extracting bioactive compounds from plant materials, including rice and other cereals [16,17]. In our previous study, this technique was applied for the first time to wheat kernels and flour using water as the extraction solvent [18]. Our initial hypothesis is that MAE extraction of phenolic compounds can provide new insight into the distribution of phenolic acids across the layers of the wheat kernel.

Given the growing interest in maximizing phenolic retention in functional foods and nutraceutical applications, the present study investigates the effect of controlled pearling intensities on phenolic acid distribution and extractability in CWRS wheat using microwave-assisted aqueous extraction. Specifically, the study integrates: (i) analysis of intact pearled kernels (PK), (ii) their derived flour fractions (PKF), and (iii) the removed pearled fractions (PF), in order to provide a comprehensive processing-based perspective on phenolic acid availability across kernel depth. The objective was not to isolate individual anatomical layers, but rather to evaluate how progressive pearling influences phenolic acid distribution and recovery within the entire kernel system.

## 2. Materials and Methods

### 2.1. Materials

Three different commercial wheat samples belonging to the Canada Western Red Spring (CWRS) class were generously provided by Cereals Canada (Winnipeg, MB, Canada), each representing a separate lot. The samples were labeled as 1, 2, and 3 to differentiate the lots. Ethanol and Folin-Ciocalteu (FC) reagent, were sourced from Fisher Scientific Canada Ltd. (Ottawa, ON, Canada). Phenolic acid standards including gallic acid, hydroxybenzoic acid, chlorogenic acid, vanillic acid, syringic acid, caffeic acid, *p*-coumaric acid, ferulic acid, sinapic acid, hydroxycinnamic acid, and salicylic acid (HPLC-grade) were obtained from Cayman Chemicals (Ann Arbor, MI, USA). Water used in the HPLC analysis was purified using a Milli-Q purification system (Billerica, MA, USA).

### 2.2. Pearling Process

The pearling procedure was performed using an abrasive-type pearling machine (Model TM-05C, Satake, Tokyo, Japan) operated at a constant rotor speed of 750 rpm. The rotational speed was maintained throughout the process using the instrument’s built-in speed control system. Pearling was conducted sequentially at predetermined time intervals (50, 100, 150, 250, 350, and 450 s). At the end of each interval, the machine was stopped and the abraded outer layer was collected and weighed. The degree of pearling was determined gravimetrically and expressed as cumulative percentage weight removal relative to the initial kernel weight, calculated as:

(1) Pearling (%) = (Weight of removed material/Initial kernel weight) × 100

The first pearling stage (50 s; PF50) removed approximately 5% of the initial kernel weight. Subsequent stages (PF100, PF150, PF250, PF350, and PF450) corresponded to cumulative removals of approximately 10%, 15%, 25%, 35%, and 45%, respectively. Thus, although samples were labelled according to pearling time (s), the actual degree of pearling was defined based on cumulative percentage weight removal.

A total of six pearled fractions (PF50-PF450) and their corresponding pearled kernel (PK50-PK450), representing the remaining kernel portions after each pearling stage, were obtained. An un-pearled control sample (PK0) was included for subsequent analysis. Continuous operational monitoring ensured stable rotor speed throughout processing. The equipment was thoroughly cleaned between pearling stages to prevent cross-contamination.

The progressive pearling approach was used as an experimental fractionation strategy to generate a phenolic distribution gradient across kernel depth and was not intended to replicate commercial milling practices.

### 2.3. Flour Attainment

The wheat kernels from each of the six fractions, along with the un-pearled wheat kernels, were ground using a cyclone mill (Udy Corporation, Fort Collins, CO, USA) equipped with a 0.5 mm screen. The attained seven pearled kernel flour (PKF) as PKF0, PKF50, PKF100, PKF150, PKF250, PKF350 and PKF450 samples were stored at −20 °C for subsequent analysis. The pearled fractions were ground using a coffee grinder (Hamilton Beach Brands Holding Company, Model 80301C, proctor silex, China) to homogenize the samples and stored at −20 °C for further analysis.

### 2.4. Moisture Content Analysis

Moisture content was determined using a moisture analyzer (Denver Instrument IR35, Denver, CO, USA) set at 130 °C for 4 min, with ten replicates used for each sample. Moisture content was determined to allow conversion of all analytical results to a dry weight basis. All reported values are expressed on a dry weight basis unless otherwise stated.

### 2.5. Microwave-Assisted Extraction

MAE of phenolic compounds from pearled fractions, pearled kernel, and the pearled kernel flour were performed using the Monowave 400 system (Anton Paar, Graz, Austria). The system’s smart vent technology ensured consistent temperature and pressure control, with a magnetic stirrer providing uniform heat distribution. Based on the preliminary optimization of the extraction conditions [18], water was used as the solvent for all samples. For the pearled kernel flour and pearled fractions, extraction was performed at 170 °C for 10 min with a solid-to-solvent ratio of 1:99. For the pearled kernel, the extraction time was extended to 15 min under the same temperature conditions, with a solid-to-solvent ratio of 1:9. This solid-to-solvent ratio was optimized in a previous study conducted in our laboratory [18]. Following extraction, the phenolic extracts were collected and centrifuged at 6000× *g* for 15 min at 4 °C using a Corning LSE centrifuge (New York, NY, USA). The supernatant was carefully extracted using a Pasteur pipette and stored at −20 °C for subsequent analysis.

### 2.6. Determination of Total Phenolic Content

The total phenolic content of the wheat fractions, including six pearled fractions and the seven pearled kernel and pearled kernel flour, was measured following a modified procedure based on [19]. A sample aliquot of 0.2 mL was added to 0.8 mL of 0.2 N Folin-Ciocalteu reagent in a 2 mL microcentrifuge tube, followed by vortexing and allowing the mixture to react for 3 min at room temperature to initiate color development. Afterward, 1 mL of sodium carbonate (Na_2_CO_3_) solution (7.5% *w*/*w*) was added to the mixture. The reaction was then kept in darkness for 30 min for full color development. The absorbance was measured at 765 nm using a microplate spectrophotometer (BioTek Epoch 2, Santa Clara, CA, USA). Ethanol served as the blank, and the calibration curve was obtained using gallic acid standards prepared in 80% ethanol at a concentration of 0.1 mg/mL to 1 mg/mL (R^2^ = 0.9992). The TPC for each sample was calculated and expressed as expressed as µg GAE/g dry weight (db).

### 2.7. Analysis of Phenolic Acids

A modified high-performance liquid chromatography-diode array detection (HPLC-DAD) method was used to analyze the phenolic acid composition of pearled fractions, pearled kernel, and pearled kernel flour, based on Shamanin et al. [20]. Samples were filtered through 0.45-μm syringe filters. Samples (20-μL injection volume) were analyzed using a reversed-phase HPLC-DAD system (Ultimate 3000, Dionex, Sunnyvale, CA, USA) at 1 mL/min flow rate. A Kinetex^®^ Biphenyl C18 100 Å RP column (2.6 μm, 150 × 4.6 mm, Phenomenex, Torrance, CA, USA) maintained at 30 °C was used for separation. A gradient elution method was employed with solvent A (water containing 0.1% acetic acid) and solvent B (acetonitrile containing 0.1% acetic acid). The gradient profile was as follows: 0–10% B (0–5 min), 10–50% B (5–20 min), 50–100% B (20–30 min), hold at 100% B until 32 min, then return to 10% B by 35 min. Chromatograms were recorded at 270 nm and 320 nm, and data analyzed using Chromeleon software (Version 7.2 SR4, Dionex Canada Ltd., Oakville, ON, Canada).

Phenolic standards including gallic acid, hydroxybenzoic acid, chlorogenic acid, vanillic acid, syringic acid, caffeic acid, *p*-coumaric acid, ferulic acid, sinapic acid, hydroxycinnamic acid, and salicylic acid were used to generate external calibration curves (Figure S1). The calibration curves exhibited high linearity, with R^2^ values ranging from 0.9762 to 0.9994, over the concentration ranges specified in Table 1. Limits of detection (LOD) and quantification (LOQ) were calculated for each standard based on a signal-to-noise ratio of 3 and 10, respectively (Table 1). These parameters ensured reliable and accurate quantification of phenolic acids in the wheat samples.

### 2.8. Statistical Analysis

All analyses were conducted using R Studio software (version 4.4.1, R Core Team, Vienna, Austria), and data expressed as means ± standard deviation from triplicate measurements. A multivariate analysis of variance (MANOVA) was used to evaluate the effects of pearling time on phenolic acid composition and total phenolic content (TPC) across pearled fractions, pearled kernels, and pearled kernel flours.

## 3. Results and Discussion

### 3.1. Kernel Morphology

**Figure 1:**
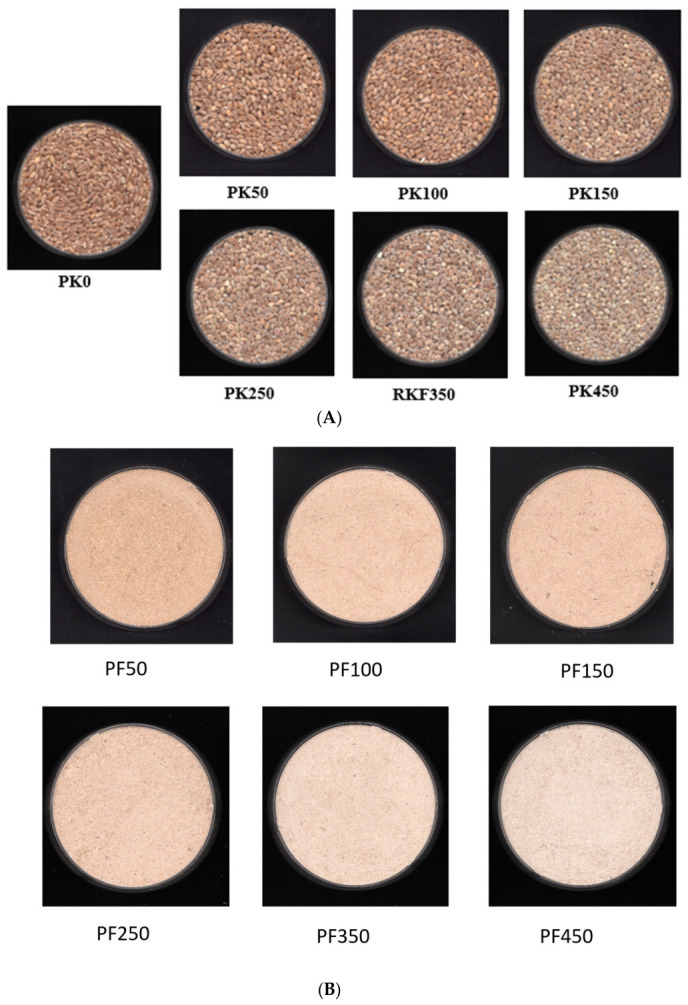
Visual representation of wheat kernels (**A**) and pearled fraction (**B**) at different pearling times. Codes: PK0: Raw whole wheat kernel; PK50: Kernels pearled for 50 s; PK100: Kernels pearled for 100 s; PK150: Kernels pearled for 150 s; PK250: Kernels pearled for 250 s; PK350: Kernels pearled for 350 s; PK450: Kernels pearled for 450 s; PF50: Pearled fraction obtained from pearling for 50 s; PF100: Pearled fraction obtained from pearling for 100 s; PF150: Pearled fraction obtained from pearling for 150 s; PF250: Pearled fraction obtained from pearling for 250 s; PF350: Pearled fraction obtained from pearling for 350 s; PF450: Pearled fraction obtained from pearling for 450 s.

The progressive change in wheat kernel morphology (PK) with increasing pearling times (50, 100, 150, 250, 350, 450 s) highlighted the gradual removal of the outer bran layers and exposure of the starchy endosperm (Figure 1A). At shorter pearling times (50 and 100 s), the kernel retained much of its original structure, with significant portions of the bran still intact. However, as pearling progressed (150 s and beyond), the outer layers, including the bran and germ, were progressively removed, resulting in a more spherical shape due to the prominence of the softer endosperm. By 450 s, most of the bran and germ were removed, leaving a kernel almost entirely composed of endosperm.

The images of the bran fractions (PF) removed during pearling are displayed in Figure 1B. A paler color was evident as the pearling process was progressing. The first bran fraction obtained from pearling the wheat kernel for 50 s was the brownest compared to the remaining fractions. In comparison, the bran from the 450 s pearling process had the lightest color due to it being higher in starchy endosperm.

### 3.2. Total Phenolic Content

**Figure 2:**
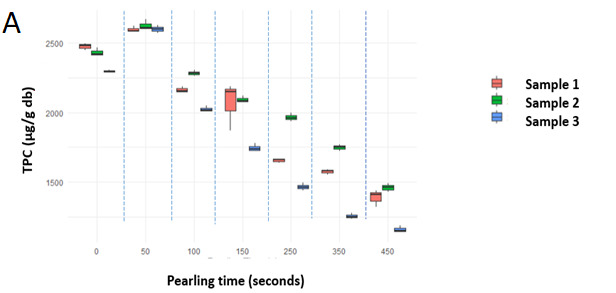

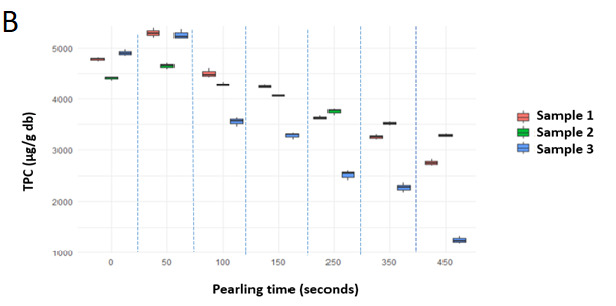

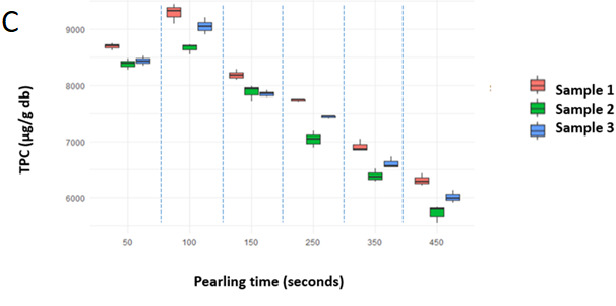
Total Phenolic Content (TPC) in (**A**). pearled kernel (PK), (**B**). pearled kernel flour (PKF), and (**C**). pearled fractions (PF), across pearling times (50, 100, 150, 250, 350, 450 s). Three different commercial wheat samples were used.

In this study, TPC analysis of the different wheat samples confirmed that as pearling progressed, from the outer layers to the inner endosperm, the TPC consistently decreased in both pearled kernel (PK) and pearled kernel flour (PKF), except for PK50 and PKF50, as shown in Figure 2A,B. Similar trend was observed in the three different samples, but the reduction of the TPC as pearling progresses was much more pronounced in the sample 3, particularly beyond 100 s pearling. Likely, the thickness of outer kernel tissues may vary within the samples, requiring shorter pearling time to reach inner kernel regions. The variation in TPC between PK and PKF across all pearling times was statistically significant (*p* < 0.05), with PKF consistently showing higher TPC than PK. The higher TPC in PKF compared to PK can be explained by the better solvent penetration and surface area in flour samples than in the kernels, enhancing the extraction of phenolic compounds, even those retained in the inner parts. A notable exception was observed with the PK50 and PKF50 fractions, corresponding to pearling at 50 s, where a higher TPC (2608 and 5065 µg GAE/g dm, respectively) was recorded compared to PK0 and PKF0 (2402 and 4699 µg GAE/g dm, respectively). The observed increase at 5% pearling may reflect structural modification of the outer kernel layers that improved phenolic extractability rather than a true increase in total phenolic concentration.

The TPC of the pearled fractions (PF) was significantly impacted by pearling time shown in Figure 2C. The MANOVA results indicated that pearling time had a significant effect on TPC (*p* < 0.05), with the highest TPC observed in PF100. While TPC generally decreased as pearling progressed, PF100, representing the 10% pearling fraction, exhibited higher TPC (9000 µg GAE/g dm) than PF50 (5% pearling fraction) (8501 µg GAE/g dm). This finding diverged from Giordano et al. [21], who observed 2883 µg/g in the 5–10% common wheat pearling fraction compared to the 3008 µg/g obtained in the 0–5% fraction. Conversely, Beta et al. previously reported that the phenolic content decreased as pearling process removes the outer layers, with the highest concentrations observed in the initial 5% of pearling fractions, which ranged from 4160 µg/g to 5300 µg/g, depending on the varieties [11]. The TPC values obtained with PKF50 for the three samples were in the higher range of those reported by Zhang et al. [13], for the bran of pigmented wheat grains (4674 to 8012 μg GAE/g) obtained by methanol-based extraction, indicating that MAE with water can effectively extract phenolic acids under the conditions applied in this study.

As pearling time increased, the subsequent pearled fractions exhibited a progressive reduction in TPC, from 7967 to 6014 µg GAE/g dm for PF150 and PF450, respectively. Therefore, the pearling process that removes the outer layers, besides the sub-aleurone region, resulted in a 29% reduction of the TPC initially present in the wheat kernel. The current results further support that pearling removes the outermost layers where phenolics are concentrated, particularly in the early stages (5–10%).

### 3.3. Phenolic Acid Profiles

#### 3.3.1. Phenolic Acid Profiles of Pearled Kernels and Flours

**Figure 3:**
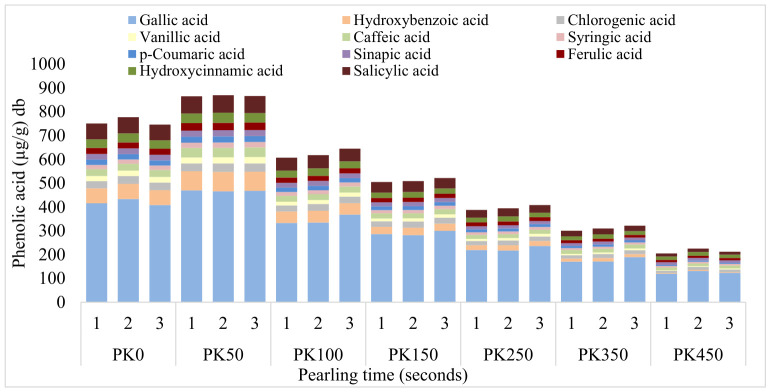
Phenolic acid profile of pearled kernel (PK) across pearling times (50, 100, 150, 250, 350, 450 s). Pearled kernel: PK0: Raw whole wheat kernel sample; PK50: Kernels pearled for 50 s; PK100: Kernels pearled for 100 s; PK150: Kernels pearled for 150 s; PK250: Kernels pearled for 250 s; PK350: Kernels pearled for 350 s; PK450: Kernels pearled for 450 s.

**Figure 4:**
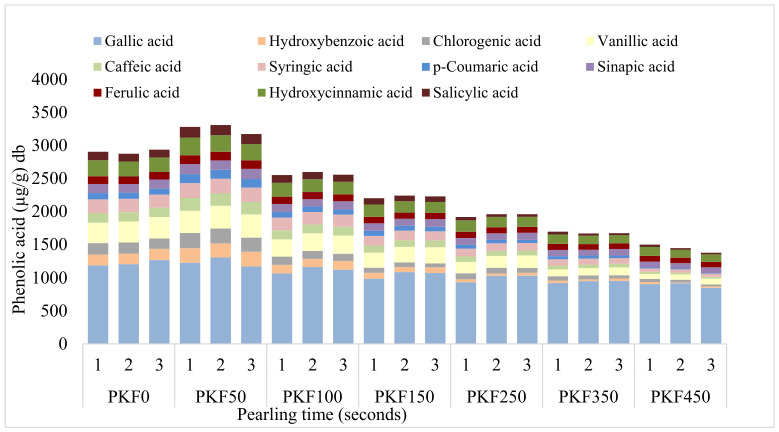
Phenolic acid profile of pearled kernel flour (PKF) across pearling times (50, 100, 150, 250, 350, 450 s). Pearled kernel flour samples: PKF0: Raw whole wheat flour sample; PKF50: Flour obtained from kernels pearled for 50 s; PKF100: Flour obtained from kernels pearled for 100 s; PKF150: Flour obtained from kernels pearled for 150 s; PKF250: Flour obtained from kernels pearled for 250 s; PKF350: Flour obtained from kernels pearled for 350 s; PKF450: Flour obtained from kernels pearled for 450 s.

The phenolic acid profiles of pearled kernels (Figure 3) and their derived flour fractions (Figure 4) were significantly influenced by pearling time. The outermost kernel tissues exhibited the highest phenolic acid content. In the kernels obtained after 50 s of pearling (PK50), gallic acid was the predominant phenolic acid extracted (~468 µg/g), suggesting its primary localization in the outer kernel tissues. Hydroxybenzoic (~81 µg/g) and salicylic acids (~73 µg/g) were the second and third most abundant phenolic acids in the outer kernel tissues. (Figure 3). In the case of salicylic acid, Verma et al. reported values for up to 11.2 μg/g among free phenolic acids in the bran of six wheat cultivars [22]. Likewise, Tian et al. identified hydroxybenzoic acid as one of the three predominant phenolic acids in pearled fractions with a content reaching up to 8.90 μg/g [16]. The significant difference in the phenolic acids content obtained in the present study confirms the efficiency of aqueous extraction using MAE even from the kernels. MAE may enhance the release of bound phenolic compounds through disruption of cell wall structures and improved solvent penetration [15].

As pearling depth increased, the total phenolic acid content progressively declined while maintaining a relatively consistent ranking of abundance (Figure 3). Gallic acid remained the most abundant, with salicylic and hydroxybenzoic acids interchanging as the second and third most prevalent. A further 50 s of pearling (from PK50 to PK100) resulted in a significant reduction in these dominant phenolic acids. Specifically, gallic acid decreased by ~36%, hydroxybenzoic acid by ~66%, and salicylic acid by ~33% indicating their predominant localization in the peripheral layers. Regarding retention after pearling, chlorogenic (~27%) and sinapic acids (~26%) exhibited the lowest loss rates in the PK100 fraction. A similar trend was observed in the 150-s fraction (PK150), where chlorogenic acid and sinapic acid exhibited losses of approximately 10 and 16%, respectively, suggesting that these acids extend beyond the outer kernel tissues into the sub-aleurone region. At 250 s of pearling, ferulic acid (~12% loss) and hydroxycinnamic acid (~16% loss) showed the lowest loss rates, suggesting a deeper distribution within the grain. In the 350-s fraction (PK350), caffeic acid (~2% loss) and sinapic acid (13% loss) exhibited the lowest losses, implying a more uniform distribution throughout the grain, including the inner layers. These cinnamic acid derivatives are likely bound as ester and/or ether linkages to cell wall components, possibly esterified to arabinoxylans or ether-linked to lignin [23]. In the final fraction (PK450), sinapic acid (~7% loss) and hydroxycinnamic acid (~14% loss) exhibited the lowest reductions, further supporting their possible association with structural components closer to storage protein compartments of the wheat kernel. This is in accordance with Ndolo and Beta [24], who reported a higher level of microwave extracted sinapic acid in the sub-aleurone region layer (245 μg/g) than in the outer kernel tissues (200 μg/g).

PKF exhibited similar trends to the kernel fractions; however, the reductions were far less pronounced, which is consistent with the substantially lower phenolic content in the endosperm (Figure 4). The highest concentrations of phenolic acids were observed in the fractions collected within the first 50 s of pearling. Gallic acid (~1234 μg/g), vanillic acid (~344 μg/g), and hydroxycinnamic acid (~255 μg/g) were the predominant compounds in this fraction. Interestingly, vanillic and hydroxycinnamic acids were not predominant in PK50, suggesting that these phenolic acids are located more internally and are therefore more effectively extracted after grinding the kernels into flour. In this study, whole kernels (PK) were subjected to MAE using water as the solvent [15]. Free phenolic acids located in the outer layers, such as the outer kernel tissues and seed coat, are expected to be readily available. Accordingly, compounds such as gallic acid, along with hydroxybenzoic and salicylic acids, predominated in the soluble fraction, as they can be easily extracted from the intact kernel structure [13]. In contrast, milling disrupted the cell walls and significantly increased the surface area, facilitating the release of phenolic acids that are otherwise bound or esterified to cell wall components such as arabinoxylans and lignin [25]. Consequently, phenolic acids such as vanillic and hydroxycinnamic acids, which are more tightly bound within the matrix, became more accessible and were extracted in higher quantities from PKF. Beyond 100 s of pearling, the overall phenolic acid content progressively declined, consistent with the trends observed in the pearled kernel (Figure 3). Among individual compounds, chlorogenic acid exhibited the greatest losses, decreasing by approximately 88% and 69% in PKF100 and PKF150, respectively. Hydroxybenzoic acid also showed substantial reductions, with decreases of approximately 70% and 56% in PKF100 and PKF150, respectively. Pearling up to 5% typically removes most of the outer kernel tissues, whereas pearling at 5–10% and 10–15% progressively removes tissues closer to the sub-aleurone region [25]. These findings suggest that chlorogenic and hydroxybenzoic acids are predominantly located in the outermost layers, between the outer kernel tissues and sub-aleurone region.

In PKF250, salicylic and hydroxybenzoic acids were among the most affected, with reductions of approximately 42% and 43%, respectively. In PKF350, vanillic and caffeic acids exhibited losses of approximately 63% and 40%, respectively. In the final fraction (PKF450), *p*-coumaric acid was almost completely depleted, with a reduction of approximately 98%. Additionally, caffeic and chlorogenic acids were markedly reduced in this fraction, with losses of approximately 95% and 52%, respectively. Since pearling for 250 and 450 s removes approximately 25–45% of the total kernel weight reaching the sub-aleurone region and the peripheral endosperm. These results suggest that salicylic, vanillic, caffeic, and *p*-coumaric acids are concentrated in those sections. Notably, in PKF450, the phenolic acid profile shifted slightly, with sinapic acid (~87 μg/g) emerging as one of the most abundant compounds. In the final fraction (PKF450), which retained approximately 55% of the initial kernel weight, the most abundant phenolic acids were gallic acid (888 μg/g), hydroxycinnamic acid (122 μg/g), sinapic acid (87 μg/g), ferulic acid (84 μg/g), vanillic acid (82 μg/g), syringic acid (49 μg/g), chlorogenic acid (39 μg/g), salicylic acid (29 μg/g), caffeic acid (28 μg/g), and *p*-coumaric acid (10 μg/g). Ndolo & Beta reported that only *p*-coumaric acid (20 μg/g) and ferulic acid (111 μg/g) were detected in wheat endosperm [24]. Therefore, MAE using water as a solvent appears to provide an effective alternative for extracting a broader range of phenolic acids from these fractions.

#### 3.3.2. Phenolic Acid Profiles of Pearled Fractions

**Figure 5:**
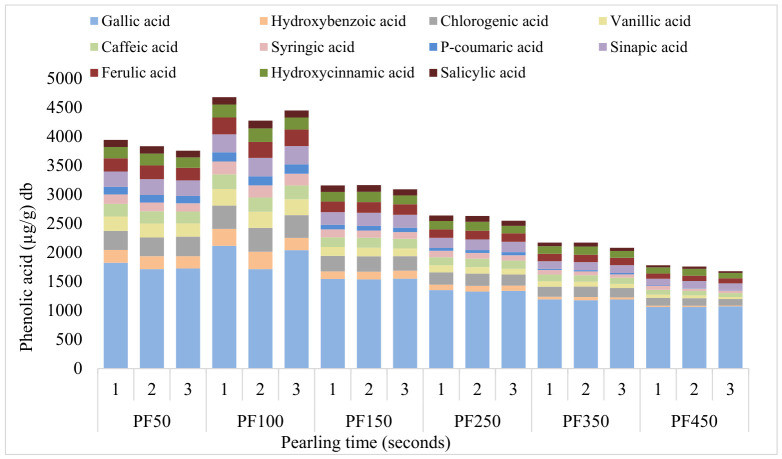
Phenolic acid profile of pearled fractions (PF) across pearling times (50, 100, 150, 250, 350, 450 s). Codes: PF50: Pearled fraction obtained from pearling for 50 s; PF100: Pearled fraction obtained from pearling for 100 s; PF150: Pearled fraction obtained from pearling for 150 s; PF250: Pearled fraction obtained from pearling for 250 s; PF350: Pearled fraction obtained from pearling for 350 s; PF450: Pearled fraction obtained from pearling for 450 s.

The phenolic acid profiles of the PF were also significantly impacted by pearling time as shown in Figure 5. In all three samples studied, the sum of phenolic acids generally decreased with increasing pearling time, except after pearling for 100-s (PF100), showing where the highest phenolic acid concentration is located. The pearled fraction PF100 showed higher concentrations of all 11 phenolic acids quantified, as shown in Figure 5, reflecting the abundance of phenolic compounds in the remaining outer kernel tissues of the wheat kernel. In contrast, the inner pearled fractions (PF350 and PF450) contained significantly lower phenolic acid concentrations, reflecting the loss of phenolic-rich layers. The reductions in individual phenolic acids, particularly those concentrated in the outer layers, were more drastic in PF150, levelling off in PF350 and PF450 at the longer pearling time, as the remaining material was predominantly endosperm.

Across all pearled fractions (50–450 s), gallic, chlorogenic, and sinapic acids consistently emerged as the most abundant phenolic compounds in pearled fractions. Gallic acid was extracted in larger amounts across all pearling times, while *p*-coumaric acid consistently showed the lowest extraction yields across all pearling fractions, with exception of PF50, PF100. Gallic (~1954 μg/g), chlorogenic (~402 μg/g), and sinapic (~314 μg/g) acids were the most abundant phenolic compounds in PF100. Barron et al. reported a sinapic acid content of 320 μg/g in water-soaked aleurone, which is in close agreement with the values obtained in this study [26]. In contrast, in the 150-s fraction (PF150), salicylic acid had the lowest loss (~15%), while gallic acid declined by roughly 27%. In fact, these were the most abundant phenolic acids in the PK pearled for 150 s, with concentrations of 289 μg/g for gallic acid and 45 μg/g for salicylic acid.

The gallic acid showed the highest retention in the later pearled fraction (PF450) indicating that while it is concentrated in the outer layers (bran and aleurone), it is still present in the sub-aleurone. Jiang et al. reported that vanillic acid (86–261 µg/g) and hydroxybenzoic acid (44–158 µg/g) were one of the most abundant phenolic acids extracted from coarse wheat bran using methanol [6]. Those values agree with the ones obtained in PF100 for vanillic acid (272–287 µg/g) and hydroxybenzoic acid (211–299 µg/g), illustrating that MAE using water is effective in extracting these phenolic acids.

## 4. Conclusions

This study highlights the critical role of pearling time in influencing the retention of phenolic compounds in wheat pearled fractions, pearled kernels and pearled kernel flours fractions. The findings underscore that shorter pearling times effectively preserve the phenolic-rich outer layers, particularly the outer kernel tissues and sub-aleurone region that account for 5–10% (*w*/*w*) of wheat kernel weight. The PF100 fraction exhibited the highest TPC among all pearled fractions, highlighting the concentration of phenolics in the outermost layers removed at the early pearling stages. The study confirmed that in the outer kernel tissues, the the predominant phenolic acids identified were gallic acid, chlorogenic acid, sinapic acid, ferulic acid, vanillic acid, hydroxybenzoic, caffeic acid, hydroxycinnamic acid, and syringic acid. In the inner kernel regions remaining after removal of the outer tissues, gallic acid, hydroxycinnamic acid, sinapic acid, ferulic acid, and vanillic acid were the most abundant. Therefore, although the different phenolic acids were present across the section of wheat kernel, chlorogenic acid appeared to be concentrated in the outer kernel tissues, whereas hydroxycinnamic acid was retained to a greater extent in inner kernel regions.

The insights gained here have practical implications for the food industry, especially in the development of functional foods increasing the phenolic content of the wheat flour by controlling milling. Future research should focus on evaluating the antioxidant properties of the fractions.

## Figures and Tables

**Table 1:** Calibration Parameters, Limits of Detection (LOD), and Limits of Quantification (LOQ) for Phenolic Acid Standards.

Phenolic Standard	Linear Range (mg/mL)	Slope (Area/mg/mL)	R^2^	Calculated LOQ (mg/mL)	Calculated LOD (mg/mL)
Gallic acid	0.05–0.8	211.8	0.9994	0.024	0.008
Hydroxybenzoic acid	0.005–0.08	529.5	0.9876	0.009	0.003
Chlorogenic acid	0.005–0.1	585.3	0.9921	0.009	0.003
Caffeic acid	0.005–0.08	866.3	0.9885	0.006	0.002
Vanillic acid	0.005–0.08	667.6	0.9902	0.008	0.003
Syringic acid	0.005–0.08	917.9	0.9850	0.005	0.002
p-Coumaric acid	0.005–0.1	1434.2	0.9867	0.003	0.001
Sinapic acid	0.005–0.06	1070.0	0.9840	0.005	0.002
Ferulic acid	0.005–0.1	1323.0	0.9875	0.004	0.0016
Hydroxycinnamic acid	0.005–0.1	389.9	0.9762	0.013	0.004
Salicylic acid	0.005–0.08	144.5	0.9789	0.035	0.012

## Data Availability

The data presented in this study are available on request from the corresponding author.

## Supplementary Materials

The following supporting information can be downloaded at: https://www.mdpi.com/article/10.3390/foods15101828/s1, Figure S1. HPLC chromatogram of eleven phenolic acid standards at two different wavelengths 270 nm (a) and 320 nm (b). Gallic acid, hydroxy benzoic acid, vanillic acid, syringic acid, and hydroxy cinnamic acid showed the highest absorption peaks at 270 nm while chlorogenic acid, caffeic acid, *p*-coumaric acid, sinapic acid, ferulic acid, and salicylic acid showed the highest absorption peaks at 320 nm.
