# Supplementary material for: Phenolic Acid Distribution in Wheat Pearling Fractions Using Microwave-Assisted Extraction
Source: Foods. 2026 May 21;15(10):1828. doi: 10.3390/foods15101828 (PMC13205254; doi:10.3390/foods15101828)
Supplement: Supplementary file 1 [file foods-15-01828-s001.zip › foods-4288038-supplementary.pdf]

Figures

S1.a

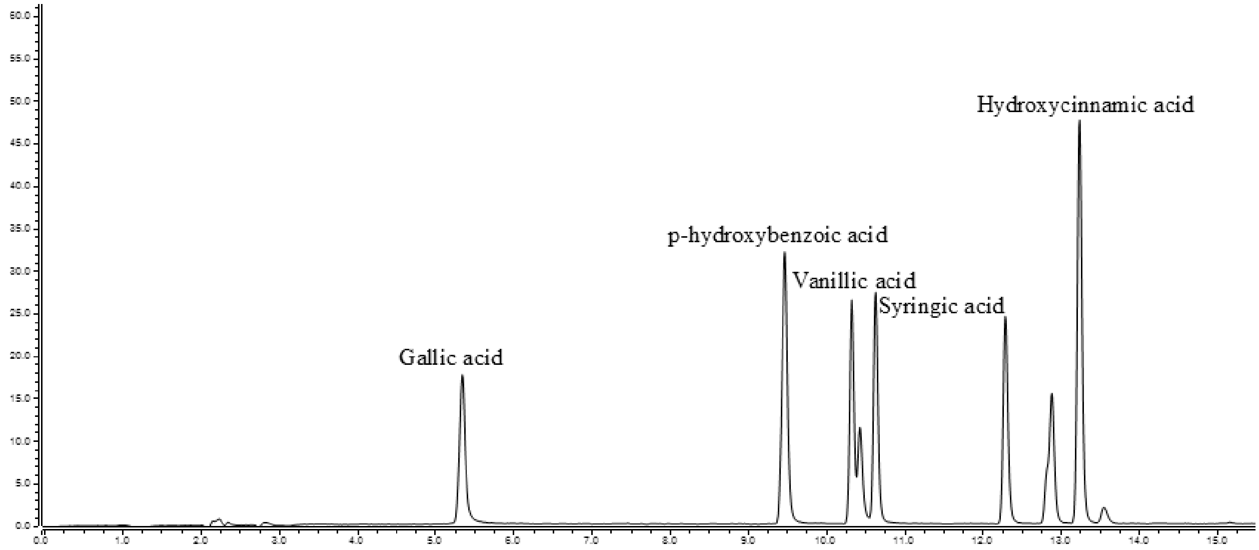

S1.b

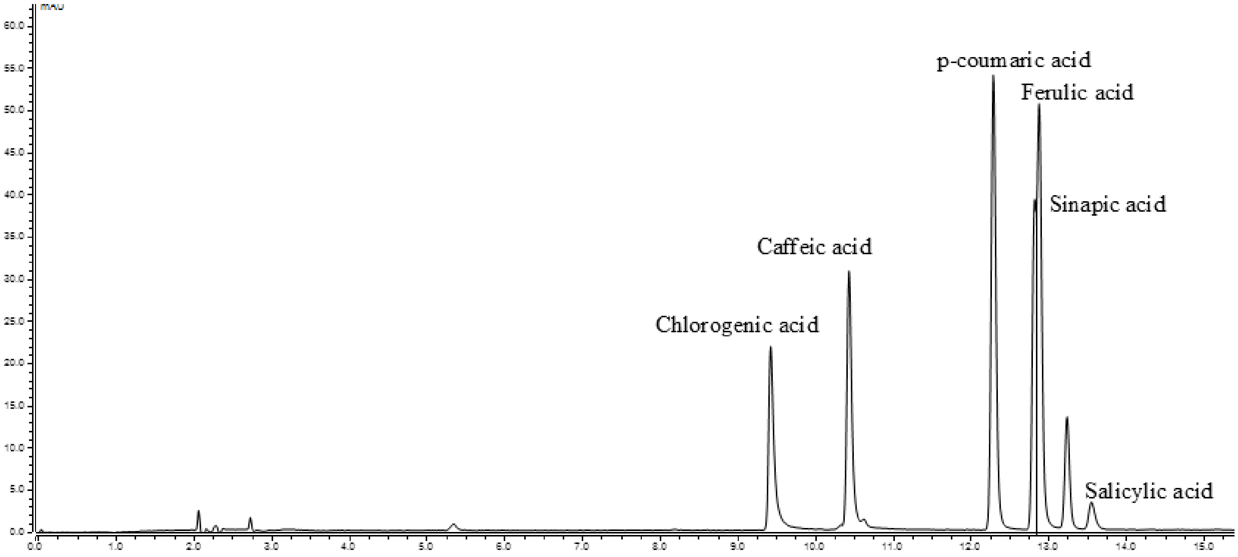

Figure S1. HPLC chromatogram of eleven phenolic acid standards at two different wavelengths 270 nm (S1.a) and 320 nm (S1.b). Gallic acid, hydroxy benzoic acid, vanillic acid, syringic acid, and hydroxy cinnamic acid showed the highest absorption peaks at 270 nm while chlorogenic acid, caffeic acid, *p*-coumaric acid, sinapic acid, ferulic acid, and salicylic acid showed the highest absorption peaks at 320 nm.
